# Non-contractability and Revenge

**DOI:** 10.1007/s10670-018-0056-y

**Published:** 2018-11-15

**Authors:** Julien Murzi, Lorenzo Rossi

**Affiliations:** University of Salzburg, Salzburg, Austria

## Abstract

It is often argued that fully structural theories of truth and related notions are incapable of expressing a nonstratified notion of defectiveness. We argue that recently much-discussed *non-contractive theories* suffer from the same expressive limitation, provided they identify the defective sentences with the sentences that yield triviality if they are assumed to satisfy structural contraction.

Here’s a standard recipe for revenge. Faced with paradoxes such as the Liar and Curry, the non-classical theorist constructs a theory of truth *S* that non-trivially expresses truth, in spite of Tarski’s Theorem. More precisely, the theorist shows that *S* can be non-trivially closed under (at least) the naïve principles Tr-R and Tr-L: 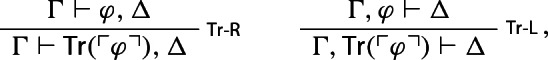


where $\ulcorner \varphi \urcorner$ is a name of $\varphi$, and $\Gamma$ and $\Delta$ range over multisets of sentences.^1^ The reason why *S* can be non-trivial is simple enough: intuitively paradoxical sentences such as the Liar sentence (a sentence asserting its untruth) don’t satisfy all the principles of classical logic in *S*, whence the paradoxical reasonings they give rise to break down. In her next step, the revenger identifies a property $\Phi$ of sentences, intuitively expressing some notion of *paradoxicality*, where a sentence $\varphi$ is paradoxical just in case absurdity follows in *S* from the assumption that $\varphi$ satisfies all the principles of classical logic. The revenger now defines a sentence $\rho$ attributing to itself the property of being $\Phi$. She then establishes via Liar-like reasoning that $\rho$ trivialises *S* if it satisfies all the principles of classical logic and that, for this reason, $\rho$ must be paradoxical, thus establishing $\rho$. But, the revenger reasons, if *S* was correctly set up, *S* only derives sentences that are not paradoxical, whence $\rho$ must be not $\Phi$. Contradiction.^2^

In this paper, we argue that a version of the strategy applies to a wide family of *non-contractive* theories, i.e. theories which reject the left and right structural rules of *contraction*: 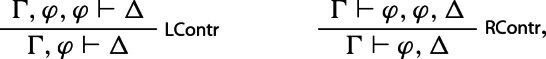


while keeping the other standard structural rules, namely *reflexivity*, *weakening* (left and right), and *cut*: 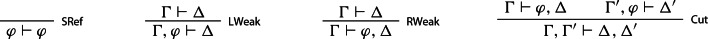


Non-contractive theories have long been advocated in the context of revisionary treatments of the semantic paradoxes, largely in virtue of their proof-theoretic elegance (see e.g. Fitch 1942, 1948). More recently, they have been claimed to be superior to standard paracomplete and paraconsistent non-classical approaches, on the grounds that, unlike them, they can handle paradoxes of naïve logical properties (Shapiro 2011b; Zardini 2015; Beall and Murzi 2013).^3^ Whatever their relative merits over standard revisionary approaches, we argue that they suffer from essentially the same expressive limitations.

Here’s our plan. Section 1 rehearses the non-contractive approach to paradox. Section 2 introduces the notions of contractability and contractable truth. Sections 3 and 4 present a revenge argument for non-contractive theories. Section 5 concludes.

## Naïve Truth, Contraction-Freedom, and Classical Recapture

Let *S* be a theory that interprets a *modicum* of arithmetic, is formulated in classical logic, and is closed under the naïve truth rules Tr-R and Tr-L. Let $\lambda$ be a sentence—a Liar sentence—provably equivalent to $\lnot {\textsf {Tr}}(\ulcorner \lambda \urcorner )$, and let negation be governed by its standard classical rules: 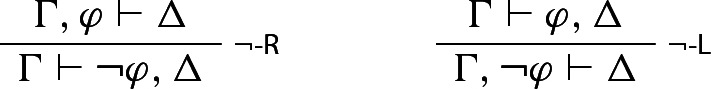


It can now be easily established that *S* is trivial.^4^ One first proves that *S* derives the sequent ${\textsf {Tr}}(\ulcorner \lambda \urcorner ) \vdash \varnothing$—call this derivation $\mathcal {D}_0$: 
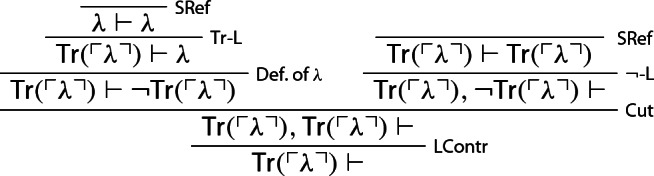


One now uses two copies of $\mathcal {D}_0$ to derive the empty sequent: 
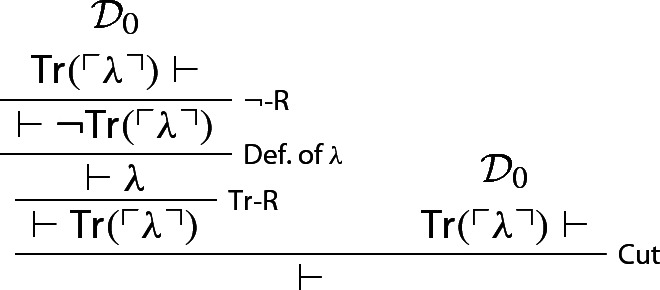


In presence of the weakening rules, every sentence is now entailed by any sentence. This is the Liar Paradox.^5^

A number of authors have recently, and not so recently, suggested blaming structural contraction as the culprit of the Liar, and of semantic paradoxes in general (Fitch 1942, 1948; Shapiro 2011b; Zardini 2011; Mares and Paoli 2014). In particular, Elia Zardini (2011) proves consistency for a non-contractive naïve theory of truth and naïve logical properties, validating naïve truth-principles such as ${\textsf {Tr}}$-R and ${\textsf {Tr}}$-L.^6^ The propositional fragment of the logic of the theory is multiplicative affine linear logic (henceforth, $\mathsf {WMLL}$)—a logic validating SRef, LWeak, RWeak, and Cut, but not $\mathsf {LContr}$ and $\mathsf {RContr}$.

Negation and the conditional are interpreted the standard way. For completeness, here are the rules for $\rightarrow$: 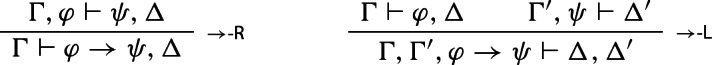


Conjunction and disjunction are interpreted, respectively, by the multiplicative connectives $\otimes$ and $\oplus$. Here are the rules for $\otimes$: 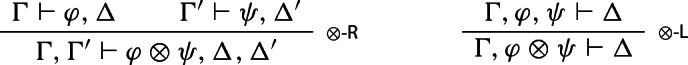


And here are the rules for $\oplus$: 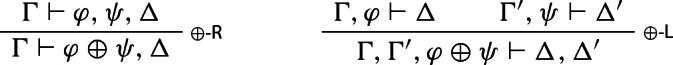


Absent SContr, the rules yield a distinctively non-classical interpretations of ‘and’ and ‘or’. For one thing, in keeping with the rejection of LContr and RContr, $\varphi$ and $\varphi \otimes \varphi$ have different logical strength: conjunction is not idempotent and $\varphi$ and $\varphi \otimes \varphi$ are not in general equivalent. For another, while $\oplus$ satisfies the Law of Excluded Middle 
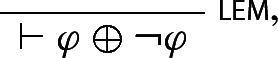


it only satisfies *weak proof by cases* (Zardini 2011, p. 516): 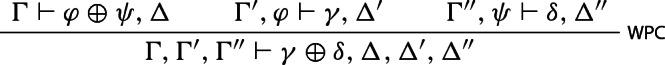


A multiplicative disjunction only entails the disjunction of whatever its disjuncts separately entail.

However, in spite of the non-classicality of $\otimes$ and $\oplus$, WMLL need not be thought as radical. In the multiplicative setting Zardini favours, full classical reasoning about $\varphi$ can be *recaptured* whenever $\varphi$ satisfies both $\varphi \rightarrow (\varphi \otimes \varphi )$ and $(\varphi \oplus \varphi ) \rightarrow \varphi$.

### Theorem 1

(Zardini 2011, Theorem 3.19) Let *S* be any theory with language $\mathcal {L}_S$ with underlying logic at least as strong as WMLL. Then, for any $\varphi \in \mathcal {L}_S$, $\varphi$ satisfies LContr and RContr if and only if it satisfies both $\varphi \rightarrow (\varphi \otimes \varphi )$ and $(\varphi \oplus \varphi ) \rightarrow \varphi$.

In particular, $\varphi \rightarrow (\varphi \otimes \varphi )$ and $(\varphi \oplus \varphi ) \rightarrow \varphi$ are, respectively, LContr- and RContr-*recapturing*, in the sense specified by the following fact:

### Fact 2

Let *S* be any theory with language $\mathcal {L}_S$ with underlying logic at least as strong as WMLL. Then, for any $\varphi \in \mathcal {L}_S$, $\varphi$ satisfies LContr if it satisfies $\varphi \rightarrow (\varphi \otimes \varphi )$ and $\varphi$ satisfies RContr if it satisfies $(\varphi \oplus \varphi ) \rightarrow \varphi$.

### Proof

The proof makes use of the following weaker versions of LContr and RContr, both of which are derivable in WMLL: 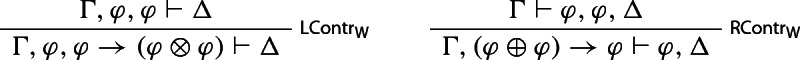


The derivability of $\mathsf {LContr}_{\mathsf {W}}$ and $\mathsf {RContr}_{\mathsf {W}}$ is respectively established by the following derivations: 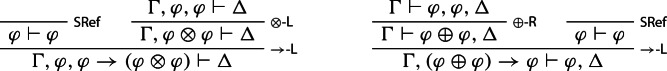


We now prove that LContr holds given $\varphi \rightarrow (\varphi \otimes \varphi )$ and $\mathsf {LContr}_{\mathsf {W}}$: 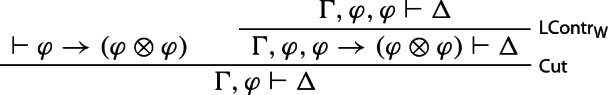


An analogous derivation establishes that RContr holds given $(\varphi \oplus \varphi ) \rightarrow \varphi$ and $\mathsf {RContr}_{\mathsf {W}}$. $\square$

## Contractability and Contractable Truth

Let WMLLTT be the result of closing a sufficiently expressive theory whose underlying logic is WMLL under Tr-R and Tr-L. Then, it is a fact about WMLLTT that sentences such as $\lambda$ satisfy LContr or RContr only on pain of triviality. That is, these sentences are *non-contractable*.^7^ Non-contractability so understood gives rise to a version of the Knower Paradox (Kaplan and Montague 1960; Myhill 1960), involving a sentence $\kappa$ provably equivalent to $\lnot {\textsf {Ct}}(\ulcorner \kappa \urcorner )$, where ${\textsf {Ct}}(x)$ is a predicate expressing contractable truth. That is, $\kappa$ says of itself that it is not true and contractable, just like the Liar sentence says of itself that it is not true. The paradox is effectively a variant of the Liar Paradox, and it is unsurprisingly invalid in non-contractive theories. However, we argue in Sect. 4 that non-contractive theorists are committed to the claim that $\kappa$ is non-contractable, which in turn triggers a version of the revenge recipe we started with.

First off, some background on contractability and related notions. Theorem 1 motivates the following rules for a contractability operator: that if $\varphi$ satisfies $\varphi \rightarrow (\varphi \otimes \varphi )$ and $(\varphi \oplus \varphi ) \rightarrow \varphi$, then $\varphi$ is contractable; and that if $\Delta$ is derivable from the assumption (represented by $\varphi \rightarrow (\varphi \otimes \varphi )$ or $(\varphi \oplus \varphi ) \rightarrow \varphi$) that one can left or right contract on $\varphi$, then $\Delta$ also follows from the assumption that $\varphi$ is contractable. In symbols, where C is an operator expressing contractability: 
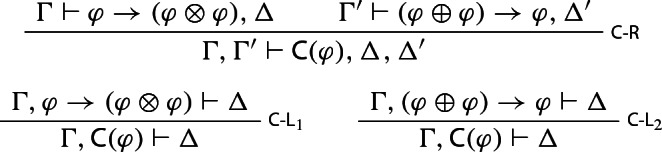


The rules can be generalised as follows. Let $[\varphi ]^{n}$ be the multiset consisting of *n* occurrences of $\varphi$. Moreover, let us assume that $\Gamma$ in ${\textsf {C}}\hbox {-}{\textsf {L}}_1^+$ and ${\textsf {C}}\hbox {-}{\textsf {L}}_2^+$ below does not contain instances of, respectively, $\varphi \rightarrow (\varphi \otimes \varphi )$ and $(\varphi \oplus \varphi ) \rightarrow \varphi$, and let $m \ge 1$. Then, one can formulate the following general rules for introducing ${\textsf {C}}(\varphi )$ on the left^8^: 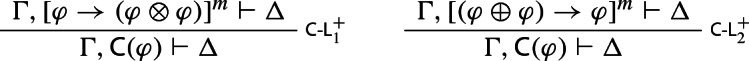


${\textsf {C}}\hbox {-}{\textsf {L}}_1^+$ says that if $\Delta$ is derivable from the assumption that $\varphi$ satisfies *m* contractions (represented by $[(\varphi \rightarrow (\varphi \otimes \varphi )]^{m}$), then $\Delta$ is derivable from the assumption that $\varphi$ is left contractable. Similarly for ${\textsf {C}}\hbox {-}{\textsf {L}}_2^+$. The rationale behind the rules is that, by non-contractive lights, structural contraction, whether left or right, is the source of the paradoxes. To see this, consider the case where $\Delta$ is the empty set in all the above left rules. Then, ${\textsf {C}}\hbox {-}{\textsf {L}}_1^+$ and ${\textsf {C}}\hbox {-}{\textsf {L}}_2^+$ say that $\varphi$ cannot be contracted on if contracting on $\varphi$, *it doesn’t matter how many times*, yields the empty set, and therefore (by weakening) *any* sentence.

To be sure, the move from $\{{\textsf {C}}\hbox {-}{\textsf {L}}_1$, ${\textsf {C}}\hbox {-}{\textsf {L}}_2\}$ to $\{{\textsf {C}}\hbox {-}{\textsf {L}}_1^+$, ${\textsf {C}}\hbox {-}{\textsf {L}}_2^+ \}$ is not altogether innocent. As a referee observed, ${\textsf {C}}\hbox {-}{\textsf {L}}_1^+$ and ${\textsf {C}}\hbox {-}{\textsf {L}}_2^+$ are derivable from ${\textsf {C}}\hbox {-}{\textsf {L}}_1$ and ${\textsf {C}}\hbox {-}{\textsf {L}}_2$ only if SContr is available. Otherwise, the best one can do (applying ${\textsf {C}}\hbox {-}{\textsf {L}}_1$ and ${\textsf {C}}\hbox {-}{\textsf {L}}_2$*m* times) is 
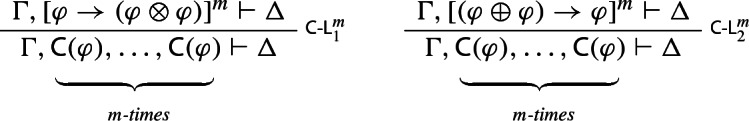


But, it might be objected, the non-contractive theorist who rejects contraction *in all its forms* has a reason to reject contracting on sentences of the form ${\textsf {C}}(\varphi )$, and hence of resting the move from $\{ {\textsf {C}}\hbox {-}{\textsf {L}}_1$, ${\textsf {C}}\hbox {-}{\textsf {L}}_2 \}$ to $\{ {\textsf {C}}\hbox {-}{\textsf {L}}_1^+$, ${\textsf {C}}\hbox {-}{\textsf {L}}_2^+ \}$.

However, ${\textsf {C}}\hbox {-}{\textsf {L}}_1^m$ and ${\textsf {C}}\hbox {-}{\textsf {L}}_2^m$ are unacceptable by non-contractive lights, since they would commit the non-contractive theorist to an untenable conception of paradoxicality. More precisely, they would commit such a theorist to distinguishing between different numbers of applications of $\mathsf {SContr}$ in a derivation, which would sit poorly with her diagnosis of what goes wrong in paradoxical derivations. According to non-contractive wisdom, *indiscriminate uses* of $\mathsf {SContr}$ must be rejected *in general*. That is, non-contractive theorists disallow the following generalised version of $\mathsf {SContr}$: 
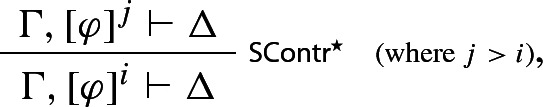


according to which, if $\Delta$ follows from $\Gamma$ and *i* occurrences of $\varphi$, then $\Delta$ follows from $\Gamma$ and at least one occurrence of $\varphi$. The idea that if $\mathsf {SContr}^{\star }$ applied to $\varphi$ leads to $\bot$ then $\varphi$ is non-contractable is at the heart of the non-contractive approach to semantic paradox: one must disallow *whatever number* of applications of $\mathsf {SContr}$ to $\varphi$ lead to $\bot$ in a paradoxical derivation. This is captured by the rules ${\textsf {C}}\hbox {-}{\textsf {L}}_1^+$ and ${\textsf {C}}\hbox {-}{\textsf {L}}_2^+$, but cannot be expressed by the non-contractive theorist who expresses non-contractability by means of rules of the form ${\textsf {C}}\hbox {-}{\textsf {L}}_1^m$ and ${\textsf {C}}\hbox {-}{\textsf {L}}_2^m$. In keeping with Fact 2, let *m*-contractions on $\varphi$ be represented by *m*-many instances of $\varphi \rightarrow (\varphi \otimes \varphi )$ or $(\varphi \oplus \varphi ) \rightarrow \varphi$). Then, the non contractive theorist who accepts ${\textsf {C}}\hbox {-}{\textsf {L}}_1^m$ and ${\textsf {C}}\hbox {-}{\textsf {L}}_2^m$ but rejects ${\textsf {C}}\hbox {-}{\textsf {L}}_1^+$ and ${\textsf {C}}\hbox {-}{\textsf {L}}_2^+$ can only express that if *m*-many contractions on $\varphi$ lead to absurdity, then *m*-many claims of the form C$(\varphi )$ lead to absurdity. In effect, this would be tantamount to introducing a denumerable infinity of contractability operators, each of which expresses *k*-contractability, for every positive integer *k*. However, it is clear that, on such a view, the non-contractive theorist would be prevented from blaming, as she does, *contraction in general* as a source of the paradoxes. Indeed, she would not be in a position to express contractability in general—in keeping with the results to be presented in Sect. 4.^9^

Now say that $\varphi$ is *contractably true* if and only if both $\varphi$ and ${\textsf {C}}(\varphi )$ hold. More formally:

$$(\mathsf {CT})$$
$$\vdash {\textsf {Ct}}(\ulcorner \varphi \urcorner ) \leftrightarrow (\varphi \otimes {\textsf {C}}(\varphi ))$$

It immediately follows that, in any theory validating $\mathsf {CT}$, contractable truth is factive:

$$(\mathsf {FACT})$$
$${\textsf {Ct}}(\ulcorner \varphi \urcorner ) \vdash \varphi $$

It can be further established that a theory *S* is closed under C-R only if it is also closed under the following necessitation-like rule:

$$(\mathsf {NEC}_{\textsf {C}})$$
If $$\Gamma \vdash \varphi , \Delta $$, then $$\Gamma , \Gamma \vdash {\textsf {C}}(\varphi ), \Delta , \Delta $$.

If $\Gamma$ and $\Delta$ are empty, $\mathsf {NEC}_{\textsf {C}}$ yields the standard rule of necessitation, that if $\vdash \varphi$, then $\vdash {\textsf {C}}(\varphi )$.

### Fact 3

Let *S* be any non-contractive theory with consequence relation $\vdash$ with underlying logic at least as strong as $\mathsf {WMLL}$. Then, *S* is closed under C-R only if it is closed under $\mathsf {NEC}_{\textsf {C}}$.

### Proof

We reason in *S*, assuming that $\varphi , \Delta$ is derivable from $\Gamma$. One first derives

$$\begin{aligned} \Gamma \vdash \varphi \rightarrow (\varphi \otimes \varphi ), \Delta \end{aligned}$$

from $$ \Gamma \vdash \varphi , \Delta $$: 
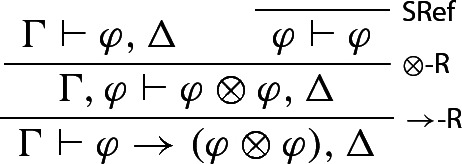


One then notices that $\Gamma \vdash (\varphi \oplus \varphi ) \rightarrow \varphi , \Delta$ is also derivable from $\Gamma \vdash \varphi , \Delta$: 
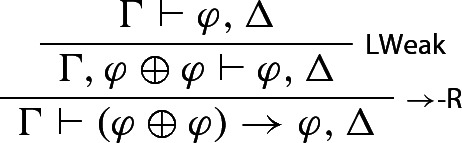


Putting the two pieces together, the sequent $\Gamma , \Gamma \vdash {\textsf {C}}(\varphi ), \Delta , \Delta$ follows by C-R.^10^$\square$

## The Non-contractability Knower

One can now generate a version of the Knower Paradox, call it the Non-contractability Knower, involving a sentence $\kappa$ provably equivalent to $\lnot {\textsf {Ct}}(\ulcorner \kappa \urcorner )$. Informally, we may reason thus. One assumes ${\textsf {Ct}}(\ulcorner \kappa \urcorner )$, derives $\kappa$ via $\mathsf {FACT}$, whence $\lnot {\textsf {Ct}}(\ulcorner \kappa \urcorner )$ by definition of $\kappa$. Assuming again ${\textsf {Ct}}(\ulcorner \kappa \urcorner )$, one must now discharge both instances of ${\textsf {Ct}}(\ulcorner \kappa \urcorner )$ and conclude $\lnot {\textsf {Ct}}(\ulcorner \kappa \urcorner )$ by $\lnot$-R . Next, one derives $\kappa$ by construction of $\kappa$, whence ${\textsf {C}}(\kappa )$ courtesy of $\mathsf {NEC}_{\textsf {C}}$. Repeating again the derivation of $\kappa$, $\kappa$ and ${\textsf {C}}(\kappa )$ now yield ${\textsf {Ct}}(\ulcorner \kappa \urcorner )$. Contradiction.

Much like in the case of the Liar, the paradox yields a result to the effect that contractable truth is undefinable in *S* if $\mathsf {LContr}$ holds.

### Definition 4

A theory *S* *defines contractable truth* if it is closed under ${\textsf {NEC}}_{\textsf {C}}$, ${\textsf {C}}\hbox {-}{\textsf {L}}_1^+$, ${\textsf {C}}\hbox {-}{\textsf {L}}_2^+$, and $\mathsf {CT}$.

While we don’t think that each of ${\textsf {NEC}}_{\textsf {C}}$, ${\textsf {C}}\hbox {-}{\textsf {L}}_1^+$, ${\textsf {C}}\hbox {-}{\textsf {L}}_2^+$, and $\mathsf {CT}$ is unassailable, they arguably jointly characterise an intuitive, if naïve, notion of non-contractability. ${\textsf {NEC}}_{\textsf {C}}$ is provable from from C-R, which is in turn justified by Theorem 1 (as shown in Fact 3). As for ${\textsf {C}}\hbox {-}{\textsf {L}}_1^+$ and ${\textsf {C}}\hbox {-}{\textsf {L}}_2^+$, we have seen in Sect. 2 that they directly fall out of the non-contractive diagnosis of the paradoxes. More precisely, when $\Gamma$ and $\Delta$ are empty, they tell us that if an arbitrary number of contractions on $\varphi$ yields triviality, then $\varphi$ is not contractable. Finally, CT simply employs the operator characterised by ${\textsf {NEC}}_{\textsf {C}}$, ${\textsf {C}}\hbox {-}{\textsf {L}}_1^+$, and ${\textsf {C}}\hbox {-}{\textsf {L}}_2^+$ to form a *predicate* expressing contractable truth. We now show that contractable truth is undefinable.

### Proposition 5

Let *S* be any theory with language $\mathcal {L}_S$ strong enough to prove the existence of a sentence $\kappa$ equivalent to $\lnot {\textsf {Ct}}(\ulcorner \kappa \urcorner )$, with underlying logic at least as strong as $\mathsf {WMLL}$. Let $\vdash$ be *S*’s consequence relation and suppose *S* defines contractable truth. Then, *S* is closed under $\mathsf {LContr}$ only if it is trivial.

### Proof

Let $\kappa$ be a sentence of $\mathcal {L}_S$ provably equivalent to $\lnot {\textsf {Ct}}(\ulcorner \kappa \urcorner )$. One first derives ${\textsf {Ct}}(\ulcorner \kappa \urcorner )\vdash$ contracting on ${\textsf {Ct}} (\ulcorner \kappa \urcorner )$—call this derivation $\mathcal {D}_1$: 
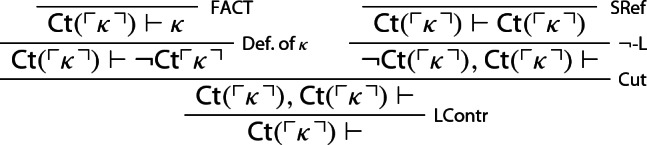


Three copies of $\mathcal {D}_1$ can now be turned into a proof of the empty sequent^11^:
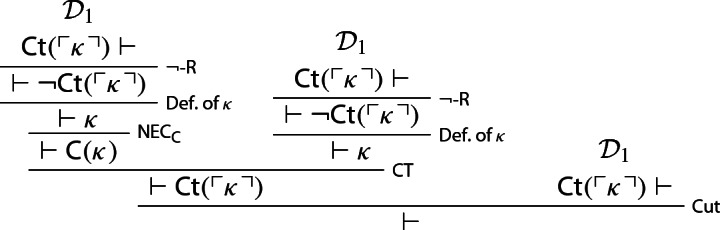


$$\square $$

This is the Non-contractability Knower.

To be sure, a natural non-contractivist response is to oserve that ${\textsf {Ct}}(\ulcorner \kappa \urcorner )$ is non-contractable, and disallow, for this reason, left contracting on ${\mathsf {Ct}}(\ulcorner \kappa \urcorner )$ in $\mathcal {D}_1$. The response is indeed available to the the non-contractive theorist, who can *prove* that ${\mathsf {Ct}}(\ulcorner \kappa \urcorner )$ is non-contractable.

### Proposition 6

Let *S* be any theory with language $\mathcal {L}_S$ strong enough to prove the existence of a sentence $\kappa$ equivalent to $\lnot {\textsf {Ct}}(\ulcorner \kappa \urcorner )$ with underlying logic at least as strong as $\mathsf {WMLL}$. Let $\vdash$ be *S*’s consequence relation and suppose *S* defines contractable truth. Then, *S* proves $\vdash \lnot {\textsf {C}}({\textsf {Ct}} (\ulcorner \kappa \urcorner ))$.

### Proof

Let $\kappa$ be a sentence of $\mathcal {L}_S$ provably equivalent to $\lnot {\textsf {Ct}}(\ulcorner \kappa \urcorner )$, and let ${\textsf {LC}}_{{\textsf {Ct}}(\ulcorner \kappa \urcorner )}$ be shorthand for

$$\begin{aligned} {\textsf {Ct}}(\ulcorner \kappa \urcorner ) \rightarrow ({\textsf {Ct}}(\ulcorner \kappa \urcorner ) \otimes {\textsf {Ct}}(\ulcorner \kappa \urcorner )). \end{aligned}$$

One first derives ${\textsf {LC}_{{\textsf {Ct}} (\ulcorner \kappa \urcorner )}}, {\textsf {Ct}}(\ulcorner \kappa \urcorner )\vdash \varnothing$—call this derivation $\mathcal {D}_2$:
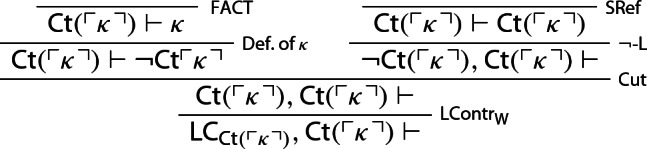


Three copies of $\mathcal {D}_2$ can now be turned into a proof that ${\mathsf {Ct}}(\ulcorner \kappa \urcorner )$ is noncontractable, courtesy of ${\textsf {C}}\hbox {-}{\textsf {L}}_1^+$:
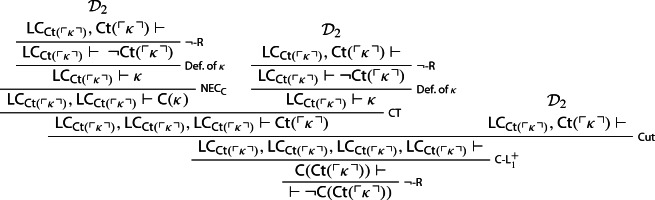
$$\square $$

Since ${\textsf {Ct}} (\ulcorner \kappa \urcorner )$ is provably non-contractable, it cannot be contracted on, and the Non-contractability Knower is blocked. If not all instances of structural contraction hold, contractable truth can be defined after all. Or can it?

## Revenge

Our argument is in two steps. We first establish that $\kappa$ is non-contractable. We then show that this very claim yields triviality.

### Lemma 7

Let *S* be any theory strong enough to prove the existence of a sentence $\kappa$ equivalent to $\lnot {\textsf {Ct}}(\ulcorner \kappa \urcorner )$, with underlying logic at least as strong as $\mathsf {WMLL}$. Then, *S* defines contractable truth only if *S* proves $\vdash \lnot {\textsf {C}}(\kappa )$.

### Proof

We notice that, by construction of $\kappa$ and the WMLL-valid rule of double negation elimination,^12^$\lnot \kappa$ entails ${\textsf {Ct}}(\ulcorner \kappa \urcorner )$, and hence $\kappa$, so that both $\kappa \vdash \kappa$ and $\lnot \kappa \vdash \kappa$ hold. Since $\kappa \oplus \lnot \kappa$ is a theorem of $\mathsf {WMLL}$, one can now infer $\kappa \oplus \kappa$ from $\kappa \oplus \lnot \kappa$, courtesy of WPC (see p. 3). More formally^13^:
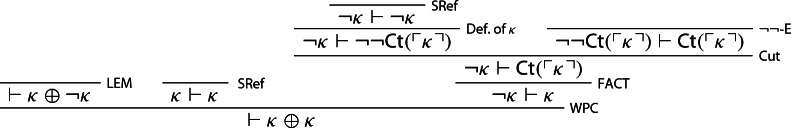


The above derivation, call it $\mathcal {D}_3$, can be used to derive $(\kappa \oplus \kappa ) \rightarrow \kappa \vdash \kappa$: 
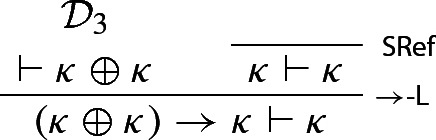


Call this derivation $\mathcal {D}_4$. We now use it to prove that $\kappa$ is non-contractable:
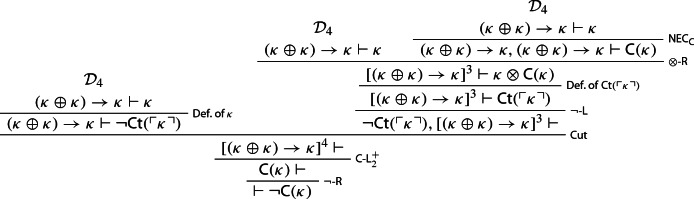
$$\square $$

The claim that $\kappa$ is non-contractable lands one into paradox once again, however. To see this, one notices in *S* that $\lnot {\textsf {C}}(\kappa )$ entails $\lnot {\textsf {Ct}}(\ulcorner \kappa \urcorner )$, and hence $\kappa$, whence $\vdash {\textsf {C}}(\kappa )$ follows, courtesy of $\mathsf {NEC}_{\textsf {C}}$. But $\kappa$ is non-contractable, i.e. $\lnot {\textsf {C}}(\kappa )$.

### Proposition 8

Let *S* be any theory strong enough to prove the existence of a sentence $\kappa$ equivalent to $\lnot {\textsf {Ct}}(\ulcorner \kappa \urcorner )$, with underlying logic at least as strong as $\mathsf {WMLL}$. Let $\vdash$ be *S*’s consequence relation and suppose *S* defines contractable truth. Then, *S* is trivial.

### Proof

Let $\kappa$ be a sentence provably equivalent to $\lnot {\textsf {Ct}}(\ulcorner \kappa \urcorner )$. Since, we’re assuming, *S* defines contractable truth, by Lemma 7*S* proves $\lnot {\textsf {Ct}}(\ulcorner \kappa \urcorner )$. We may then reason thus^14^: 
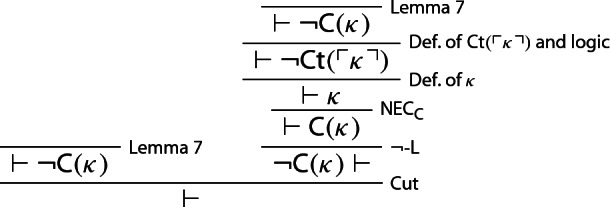
$$\square $$

This is bad news for the non-contractive theorist. Lemma 7 establishes that *S* defines contractable truth only if it proves $\lnot {\textsf {C}}(\kappa )$. But it follows from Proposition 8 that any such *S* proves $\lnot {\textsf {C}}(\kappa )$ only if it is trivial.

## Concluding Remarks

Our argument requires five main ingredients:

(i) $$\mathsf {FACT}$$, to get $$\vdash \kappa \oplus \kappa $$;
(ii) that $\vdash \kappa$ and $\vdash {\textsf {C}}(\kappa )$ yield $\vdash {\textsf {Ct}}(\ulcorner \kappa \urcorner )$;
(iii) that *S* be closed under ${\textsf {C}}\hbox {-}{\textsf {L}}_2^+$, to get $\vdash \lnot {\textsf {C}}(\kappa )$;
(iv) that $\lnot {\textsf {C}}(\kappa ) \vdash \lnot {\textsf {Ct}}(\ulcorner \kappa \urcorner )$, to turn the claim that $\kappa$ is non-contractable into an assertion of $\kappa$ itself;
(v) that $\lnot {\textsf {C}}(\kappa )$ yields triviality.

Items (i), (ii), and (iv) come straight from CT, the definition of ${\textsf {Ct}}(\ulcorner \kappa \urcorner )$, and some basic features of the logic WMLL. Item (iii) is motivated by the assumption that a sentence is non-contractable in *S* if and only if contracting on it in *S* (it doesn’t matter how many times) makes *S* trivial. As for the claim that $\lnot {\textsf {C}}(\kappa )$ yields triviality, namely (v), it turns on $\mathsf {NEC}_{\textsf {C}}$. But, as Fact 3 shows, $\mathsf {NEC}_{\textsf {C}}$ is derivable from C-R, which is in turn justified by Theorem 1.

To be sure, we’ve assumed throughout that any adequate semantic theory should be non-hierarchical, in the sense of being able to consistently express meta-theoretical notions such as non-contractability. However, we submit, expressing in the object-language notions that have been traditionally formalised in a meta-theory is an integral part of the effort of treating truth and other fundamental semantical and logical concepts in one single language (Reinhart 1986, pp. 227–229; Field 2008, p. 18). We conclude that the theory of contractable truth for a contraction-free theory *S* cannot be formulated in *S*.

## Footnotes

1 Multisets are just like sets, except that repetitions count. For instance, $\{a, a\}$ and $\{a\}$ are the same set, but [*a*, *a*] and [*a*] are different multisets (we represent multisets by means of square brackets).

2 See Priest (2007, p. 226). For recent discussion on revenge, see e.g. Beall (2007b), Shapiro (2011a) and Scharp (2013). We should note that the revenge recipe just sketched only applies to consistent theories. However, it can be modified so as to also cover inconsistent approaches (see Murzi and Rossi 2018).

3 For general background on paracomplete approaches, see e.g. Kripke (1975), Field (2007, 2008) and Horsten (2009); on paraconsistent approaches, see e.g. Asenjo and Tamburino (1975), Goodship (1996), Priest (2006) and Beall (2009, 2011). We should also note that, among non-contractive theorists, Elia Zardini has explicitly acknowledged that the handling of the paradoxes of naïve logical properties comes with what may be regarded as a cost, viz. that the meta-theory must itself be non-classical, and indeed substructural (Zardini 2013, 2014). We return to this point in Sect. 2 below.

4 We implicitly make use of a rule of intersubstitutivity of equivalents for sentences, here and throughout. Nothing crucial hinges on this choice.

5 We note that the present version of the Liar does not rely on the Law of Excluded Middle or similar classical principles.

6 Zardini’s theory actually validates a much stronger form of naïveté, viz. the intersubstitutivity *salva veritate* of $\varphi$ and ${\textsf {Tr}}(\ulcorner \varphi \urcorner )$ in all non-opaque contexts.

7 How can a restriction of contraction be plausibly motivated? As John Myhill once put it, ‘while [restricting contraction] is *proof-theoretically* natural [...] no form known to us is *philosophically* natural’ (Myhill 1975 p. 182). In a number of papers, Zardini has recently sought to justify restrictions of LContr and RContr on the grounds that sentences such as $\lambda$ are unstable where, according to Zardini, a sentence $\varphi$ is unstable if and only if there is a $\psi$ such that the state-of-affairs expressed by $\varphi$ leads to the state-of-affairs expressed by $\psi$ and those two states-of-affairs are incompatible—see Zardini (2015, p. 492) and, especially, Zardini (2011, pp. 503–506). But how to more precisely interpret Zardini’s notion of instability? As Zardini puts it, the notion of an unstable state-of-affairs involves stepping ‘out of the abstract realm of *formal* theories of truth $\dots$ [to] engage in some concrete *metaphysics* of truth’ (Zardini 2011, p. 504). Here we briefly note that, from a purely logical point of view, Zardini’s notion of instability is difficult to make precise. It might be understood meta-theoretically as the claim that, for some suitable non-contractive theory *S* with language $\mathcal {L}_S$, $\varphi \vdash _S \psi$ but $\not \vdash _S \varphi \otimes \psi$. But such a reading would appear to be too strong. For suppose *S* interprets a *modicum* of arithmetic and let $\gamma$ be a Gödel sentence for *S*. Now consider one of $\gamma$’s consequences, such as $\gamma \oplus \gamma$. Then, since $\not \vdash _S \gamma$, it follows that $\not \vdash _S \gamma \otimes (\gamma \oplus \gamma )$ also holds. And since $\gamma \vdash _S \gamma \oplus \gamma$, the sentence $\gamma$ winds up being unstable. Yet this appears to be problematic: if we let *S* be Peano Arithmetic, $\gamma$ is an arithmetical truth! Alternatively, it might be thought that $\varphi$ is unstable if and only if $\varphi \vdash _S \psi$ and $\varphi , \psi \vdash _S$. But such a reading is also too strong. Since $\lnot t=t \vdash _S t=t$ and $\lnot t=t, t=t \vdash _S$, it follows that $\lnot t=t$ also winds up being unstable. Yet, intuitively, $\lnot t=t$ is simply false, since it is false in every model validating the classical theory of identity, and would rather appear to be stable. For these reasons, in constructing a revenge argument for non-contractive approaches to paradox, we focus on the minimal notion of non-contractability discussed in the main text.

8 A related principle is used in (Murzi and Rossi 2018) to run a different argument against non-contractive approaches.

9 We are grateful to Lionel Shapiro and an anonymous referee for valuable discussion on this point.

10
Beall (2006) suggests that the intuitive defectiveness of paradoxical sentences may be expressed by an operator ${\textsf {PN}}$ expressing *paranormality*. He then notices that one could define a factive notion of robust truth $\mathbb {T}(x)$ by setting $\mathbb {T}(\ulcorner \varphi \urcorner ) \leftrightarrow (\varphi \wedge \lnot {\textsf {PN}}(\varphi ))$. However, he also insists that $\mathbb {T}(x)$*may not satisfy necessitation*. That is, one should not expect that, if $\vdash \varphi$, then $\vdash \mathbb {T}(\ulcorner \varphi \urcorner )$. As a result, some paranormal sentences are true, ‘but not thereby robustly true’ (Beall 2007a, §4.2). It is a consequence of Fact 3 that Beall’s strategy doesn’t apply to theories closed under C-R.

11 The line labelled ‘CT’ abbreviates a Cut applied to $\vdash \kappa \otimes {\textsf {C}}(\kappa )$ and $\kappa \otimes {\textsf {C}}(\kappa ) \vdash {\textsf {Ct}}(\ulcorner \kappa \urcorner )$, which follows from the right-to-left direction of the schema CT of p. 6, namely $\vdash {\textsf {Ct}}(\ulcorner \varphi \urcorner ) \leftrightarrow (\varphi \otimes {\textsf {C}}(\varphi ))$.

12 See Zardini (2011, Theorem 3.8, p. 514).

13 The lines labelled ‘LEM’ and ‘$\lnot \lnot \hbox {-}{\textsf {E}}$’ respectively abbreviate WMLL-proofs of $\kappa \oplus \lnot \kappa$ and $\lnot \lnot {\textsf {Ct}}(\ulcorner \kappa \urcorner ) \vdash {\textsf {Ct}}(\ulcorner \kappa \urcorner )$.

14 The line labelled ‘Def. of ${\textsf {Ct}}(\ulcorner \kappa \urcorner )$ and logic’ abbreviates the following passages: from $\lnot {\textsf {C}}(\kappa )$ to $\lnot {\textsf {C}}(\kappa ) \oplus \lnot \kappa$ (by right weakening and $\oplus \hbox {-}{\textsf {R}}$), then from $\lnot {\textsf {C}}(\kappa ) \oplus \lnot \kappa$ to $\lnot ({\textsf {C}}(\kappa ) \otimes \kappa )$ (by the DeMorgan laws, which hold in WMLL), and finally from the latter to $\lnot {\textsf {Ct}}(\ulcorner \kappa \urcorner )$ by definition of Ct, i.e. the schema CT of p. 6 (see also footnote 11).
